# A Genetic Predictive Model for Canine Hip Dysplasia: Integration of Genome Wide Association Study (GWAS) and Candidate Gene Approaches

**DOI:** 10.1371/journal.pone.0122558

**Published:** 2015-04-13

**Authors:** Nerea Bartolomé, Sergi Segarra, Marta Artieda, Olga Francino, Elisenda Sánchez, Magdalena Szczypiorska, Joaquim Casellas, Diego Tejedor, Joaquín Cerdeira, Antonio Martínez, Alfonso Velasco, Armand Sánchez

**Affiliations:** 1 Progenika Biopharma SA, a Grifols Company, Derio, Bizkaia, Spain; 2 Bioibérica SA, Barcelona, Spain; 3 Servei Veterinari de Genètica Molecular, Departament de Ciència Animal i dels Aliments, Facultat de Veterinària, Universitat Autònoma de Barcelona, Barcelona, Spain; 4 Centro Veterinario Aluche Las Águilas, Madrid, Spain; Tor Vergata University of Rome, ITALY

## Abstract

Canine hip dysplasia is one of the most prevalent developmental orthopedic diseases in dogs worldwide. Unfortunately, the success of eradication programs against this disease based on radiographic diagnosis is low. Adding the use of diagnostic genetic tools to the current phenotype-based approach might be beneficial. The aim of this study was to develop a genetic prognostic test for early diagnosis of hip dysplasia in Labrador Retrievers. To develop our DNA test, 775 Labrador Retrievers were recruited. For each dog, a blood sample and a ventrodorsal hip radiograph were taken. Dogs were divided into two groups according to their FCI hip score: control (A/B) and case (D/E). C dogs were not included in the sample. Genetic characterization combining a GWAS and a candidate gene strategy using SNPs allowed a case-control population association study. A mathematical model which included 7 SNPs was developed using logistic regression. The model showed a good accuracy (Area under the ROC curve = 0.85) and was validated in an independent population of 114 dogs. This prognostic genetic test represents a useful tool for choosing the most appropriate therapeutic approach once genetic predisposition to hip dysplasia is known. Therefore, it allows a more individualized management of the disease. It is also applicable during genetic selection processes, since breeders can benefit from the information given by this test as soon as a blood sample can be collected, and act accordingly. In the authors’ opinion, a shift towards genomic screening might importantly contribute to reducing canine hip dysplasia in the future. In conclusion, based on genetic and radiographic information from Labrador Retrievers with hip dysplasia, we developed an accurate predictive genetic test for early diagnosis of hip dysplasia in Labrador Retrievers. However, further research is warranted in order to evaluate the validity of this genetic test in other dog breeds.

## Introduction

Canine hip dysplasia (CHD) is one of the most prevalent developmental orthopedic diseases in dogs worldwide. It is characterized by an abnormal formation of the hip joint with different degrees of laxity and subluxation, which ultimately leads to secondary osteoarthritis (OA) and impaired animal welfare. The prevalence of CHD is particularly high among larger breeds of dogs, estimates of around 20% have been found in Labrador retrievers, one of the most popular breeds in the world, and up to 70% in Saint Bernards [1–3].

The diagnosis of CHD is established through radiographic examination of the hip joint using different accepted scores. The FCI [Fédération Cynologique Internationale] scale is one of the most popular in Europe, while the OFA [Orthopedic Foundation for Animals] hip score is the most commonly used in USA [4]. During the last decades a high number of selective breeding programs based on radiographies have been implemented for different breeds with the aim of reducing CHD incidence and improving animal’s welfare. However, the phenotype-based screening programs have not been effective enough, since the prevalence of CHD remains high. An improvement was described in some cases [5,6], but a slow progress or no improvement was achieved in others [7–9].

Current knowledge in the mode of inheritance of CHD indicates it to be a genetic complex trait with a polygenic inheritance pattern influenced by environmental factors. Both dominant and recessive modes of inheritance have been proposed [10–13]. The results of several studies performed in the last five years suggest that selective breeding programs based on genetic information, rather than on phenotypic selection alone, are the best alternative to achieve more rapid improvements in CHD [14–16].

Molecular genetic studies with microsatellites led to the identification of quantitative trait loci [QTL] for CHD and secondary OA in different breeds, such as Labrador retrievers [17,18] or Portuguese water dogs [19,20] some years ago. The sequencing of the whole dog genome and the characterization of more than 2.5 million single nucleotide polymorphisms [SNPs] in 2005 [21] opened the door to the development of new genotyping tools, as high-throughput SNP genotyping microarrays and, thus, to new studies aimed at clarifying the genetic basis of CHD. The usefulness of predictive models based on combinations of SNPs, as genetic markers, for assessing susceptibility to specific diseases has been long demonstrated in humans [22–24]. The first published studies using SNPs as genetic markers for CHD have generated interesting and promising results. A group of SNPs which confers increased risk for CHD has been identified in German shepherd dogs [25]. A Genome Wide Association Study [GWAS] with more than 22,000 SNPs in dogs of several breeds found 4 SNPs associated to CHD and 2 SNPs to hip OA [26]. Besides, association studies with SNPs have allowed redefining QTL intervals and identification of the first mutation associated with canine hip dysplasia, which is located in the fibrillin-2 gene [*FBN2*] [27–29]. Finally, a very recent GWAS performed with nearly 18,000 SNPs and with Labrador retrievers has identified several SNPs in genes or near genes involved in extracellular matrix development associated to CHD [30]. A second GWAS with around 47,000 SNPs, published during the writing of this manuscript, has identified several SNPs associated to CHD in German Shepherd Dogs [31].

Recently, a new commercial high density SNP genotyping microarray [Canine HD BeadChip, Illumina Inc, San Diego, CA, USA] with a genome wide coverage [170,000 SNPs evenly spaced in the dog genome] has become available at the market. The goal of the present study is to find a predictive model based on genetic markers for CHD susceptibility in Labrador retrievers. For that purpose, we have performed a GWAS using the Canine HD BeadChip and a candidate gene study in which hundreds of SNPs in several candidate genes related to inflammation, bone formation and cartilage remodeling pathways, among others, were analyzed. We have developed a genetic predictive model, based on 7 SNPs, able to predict CHD development in Labrador retrievers.

## Materials and Methods

### Ethics Statement

The study was approved by the Ethics Committee on Animal and Human Experimentation (CEEAH) of the Universitat Autònoma de Barcelona (UAB, Spain) (Authorization reference number: DMAH 4463). All animal work has been conducted according to the national and international guidelines for animal welfare. All blood-sampling was done in veterinary clinics for small animals and with the owners' informed consent.

### Study population and X-ray evaluation

A total of 775 pure breed Labrador retriever dogs (633 for the development study and 142 for the validation) were recruited from 64 Spanish veterinary clinics. Both the Labrador retriever show and field lines were equally represented in this study, including dogs from European (British and continental) and American bloodlines. This was verified by using a specific pedigree software (Breeders Assistant for dogs 4, Tenset Technologies Ltd., Cambridge, UK). Regarding inbreeding rates, the animals were unrelated at least at the grandparent level.

A standard ventrodorsal hip X-ray of each dog was taken. All the X-rays were independently evaluated by three experts from the Hip Dysplasia Radiological Reading Committee at the Spanish Small Animal Veterinary Association (AVEPA) who were unaware of the dogs' genotypes. X-rays were evaluated according to the FCI (Fédération Cynologique Internationale) official scale for hip dysplasia (A = no signs of CHD, B = near normal hips, C = mild signs of CHD, D = moderate signs of CHD, E = severe CHD). The FCI grade should be coincident in the view of at least two of the three evaluators and should not differ from the evaluation of the third evaluator in more than 1 grade in the FCI scale. In case these criteria were not met, radiographs were re-evaluated until the criteria were fulfilled.

We followed an extreme phenotype design for the association analysis, so C dogs were not included in the sample. Dogs scored as A or B were classified into the control group (free of hip dysplasia) and dogs scored as D or E were classified into the case group (affected). To be sure that the dogs classified as A or B had developed a final phenotype, and will not evolve to other grades, a minimum age of 12 and 48 months was required for A and B dogs, respectively. No minimum age was required for dogs scored as D and E, although a maximum of 8 years old was set in order to avoid the interference of osteoarthritic changes due to aging. Among the 775 dogs 354 fulfilled the inclusion criteria and were finally genotyped (240 for the development study and 114 for the validation).

### DNA extraction and SNP genotyping

Blood samples were collected in EDTA-tubes. The genomic DNA was extracted using the QIAamp DNA blood mini kit (Qiagen, Hilden, Germany) following the manufacturer instructions. Purified DNA was quantified using Qubit fluorometer (Life Technologies-Molecular Probes, Oregon, USA) following manufacturer instructions or Nanodrop spectrophotometer (Thermo Fisher Scientific, Wilmington, DE, USA) and adjusted to the desirable concentration. The A260/280 ratio was above 1.6 for all samples.

We combined two different strategies for identification of SNPs associated to canine hip dysplasia in the development population. On one hand, we performed a Genome Wide Association Study (GWAS) using the Canine HD BeadChip (Illumina Inc, San Diego, CA, USA) which analyzes more than 170,000 evenly spaced SNPs in the dog genome. On the other hand, we genotyped 768 custom SNPs located in candidate genes and quantitative trait loci (QTL) for CHD with the Illumina Golden Gate genotyping platform (Illumina Inc, San Diego, CA, USA). We selected as candidate genes, genes implicated in molecular processes involved in CHD and/or OA (cartilage degradation, inflammation, extracellular matrix metabolism and bone remodeling, among others), genes previously described as being associated with OA in humans, genes involved in cartilage and bone diseases in humans and genes located in QTLs previously reported as associated with CHD or OA [17, 19, 20, 28, 32]. We used dbSNP (http://www.ncbi.nih.gov/projects/SNP) and CanFam (http://www.broadinstitute.org/mammals/dog) databases for SNPs selection. We chose 2 or 3 SNPs per gene, selecting intragenic SNPs, when possible.

The dogs of the validation population were genotyped for the SNPs of interest using the KASPar chemistry (LGC Genomics, Hertfordshire, UK), which is a competitive allele-specific PCR SNP genotyping system that uses FRET (Förster Resonance Energy Transfer) quencher cassette oligonucleotides.

### CHST3 sequencing and analysis

A chromosomal fragment of 5819 bp including the *CHST3* gene and its 5’ upstream and 3’ downstream flanking regions was sequenced by Sanger method in 39 Labrador retrievers (20 unaffected individuals and 19 with hip dysplasia) using as reference the Boxer sequence of the *CHST3* gene (NCBI GeneID: 489036; build 2.1). Six overlapping PCRs were performed using the Qiagen Multiplex PCR kit (Qiagen, Hilden, Germany), with an annealing temperature of 60°C and 100 ng of DNA template. PCR products were purified using Millipore HTS filter plates (Merck Millipore, Darmstadt, Germany). Sequencing reactions were performed with BigDye Terminator v3.1 Cycle Sequencing kit (Applied Biosystems, Carlsbad, CA, USA). Samples were cleaned with CleanSEQ reaction clean-up (Agencourt Bioscience Corp., Beverly, MA, USA) and analyzed on an ABI 3100 DNA Analyzer.

### Statistical analysis

Monomorphic single nucleotide polymorphisms, SNPs with MAF less than 0.01, SNPs with a call rate <99% and SNPs with severe deviation from Hardy-Weinberg equilibrium in controls (p<0.0001) were excluded from the analyses, both in the GWAS and in the candidate gene analyses. This filtering resulted in the selection of 449 SNPs from the candidate gene study and 130,621 SNPs from the GWAS. Principal component analysis (PCA) was carried out to assess for population stratification of the development study cohort (240 individuals). 80,116 SNPs remained after quality control, LD pruning and inactivation of the X chromosome, and were used for the PCA. Analyses were performed using the SNP & Variation Suite (SVS v7.0) software of Golden Helix.

An association test for SNP allele frequencies with CHD was performed by the chi-square test in the GWAS and the candidate genes studies. To adjust for multiple testing, we used the Benjamini-Hochberg procedure to control False Discovery Rate (FDR) [33]. Based on the multiple test correction made in our data set, the minimum uncorrected p-value threshold that would reach statistical significance (at the 5% level) after correction for FDR would be p<1.96x10^-5^ in the GWAS analysis and p<5x10^-3^ in the candidate gene study. We developed a multivariate predictive model for CHD by means of forward logistic regression using as predictors the SNPs significant after controlling for FDR. We tested our model discrimination via the Hosmer_Lemeshow statistic and the receiver operating characteristic (ROC) curve with 95% CIs. To measure the impact of the SNPs included in the model the sensitivity (Se) and specificity (Sp) values were computed by means of the ROC curve.

The accuracy of the predictive model was tested on the independent validation cohort. A ROC curve was constructed with the predicted probability score for the model generated by the equation derived in the development cohort for each subject of the validation cohort. The statistical difference between the AUCs of the ROC curves of the two cohorts was calculated by means of a two-sample Z-test.

To determine the contributing chi-square value of each predictor, the predictive model was calculated by eliminating one predictor at a time. The chi-square value for the reduced model was recorded for each factor. The chi-square value of each factor was subtracted from the chi-square value of the full model to determine its percentage contribution [34].

The univariate statistical analysis was performed with the softwares SVS v7.3.1 (Golden Helix Inc.) for the candidate gene study and PLINK v1.07 for the GWAS. The software SPSS v15.0 was used for the multivariate analysis. Measures of pairwise linkage disequilibrium (LD) (r^2^>0.8) were determined using SVS v7.3.1 software (Golden Helix Inc.).

## Results

**Fig 1:**
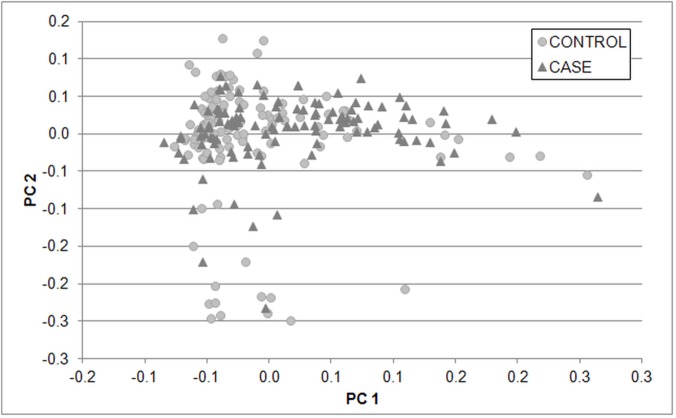
Principal Component Analysis (PCA) plot of the study development cohort. 80,116 SNPs dispersed genome-wide were used. X-axis is principle component 1 and y-axis is principle component 2. Cases and controls were clustering together.

PCA plot of the 240 Labrador included at the development study and 80,116 SNPs revealed no stratification among the affected dogs and free of hip dysplasia dogs (Fig 1). The distribution according to the FCI scale for hip dysplasia and the average age of the Labrador retrievers included in the development (n = 240) and validation (n = 114) cohorts are depicted in Table 1.

**Table 1:** Distribution according to the FCI scale for hip dysplasia and average age of the Labrador retrievers included in the development and validation cohorts.

		**Development cohort**		**Validation cohort**	
	FCI grade	n	Age (mean ± SD)	n	Age (mean ± SD)
**Controls**	A	99	34 ± 18	44	21 ± 12
	B	30	64 ± 11	14	64 ± 24
**Cases**	D	64	37 ± 21	26	48 ± 37
	E	47	45 ± 28	30	48 ± 48

**Fig 2:**
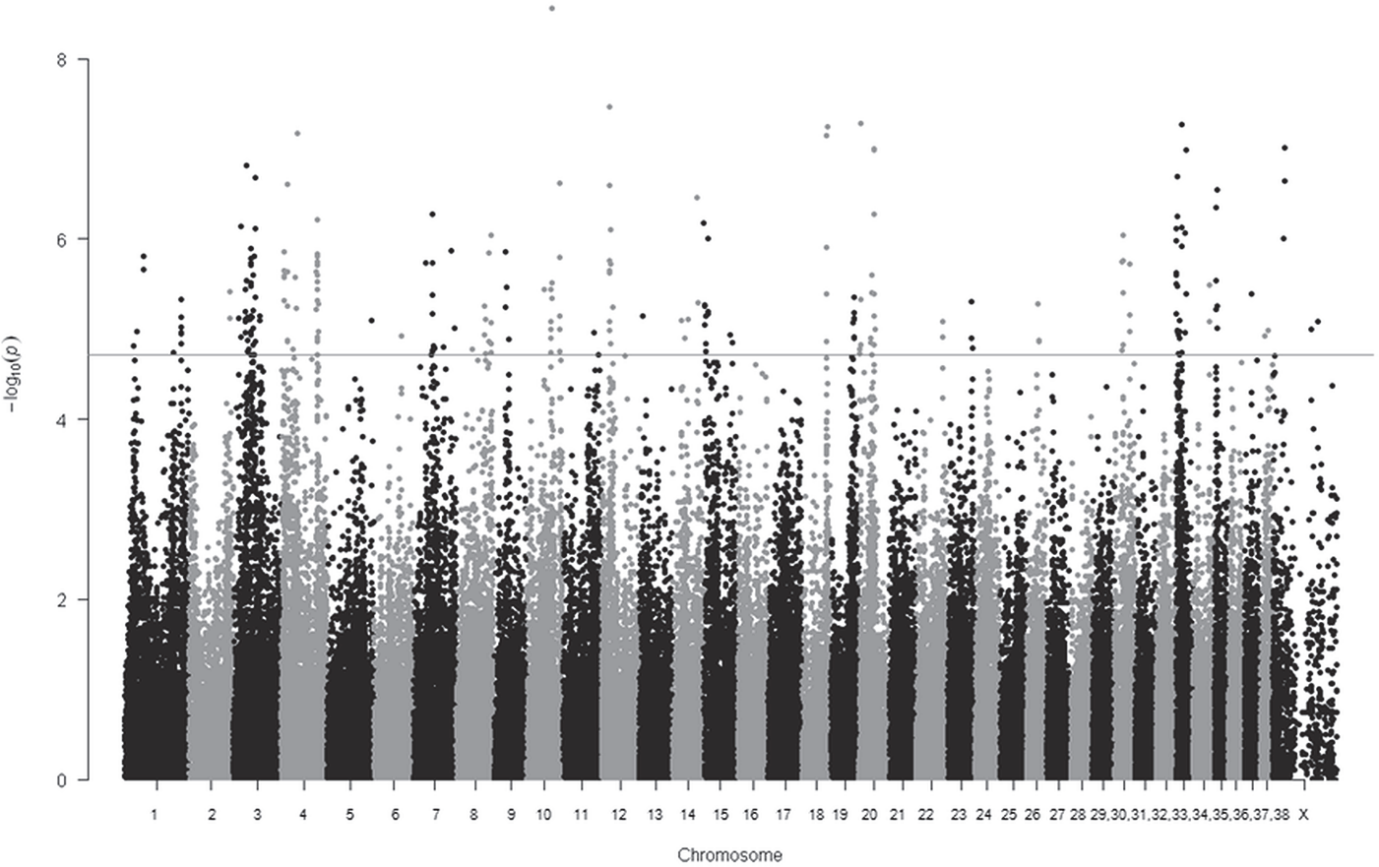
Manhattan plot for the CHD association test results obtained in the GWAS. The horizontal line indicates the FDR correction significance level (p≤1.96x10^-5^).

In the development cohort, we found 250 SNPs significantly associated to CHD after FDR correction for multiple testing in the GWAS analysis (p≤1.96x10-5) and 33 SNPs in the candidate genes study (p≤5x10-3) (Fig 2, S1 Table and S2 Table).

**Fig 3:**
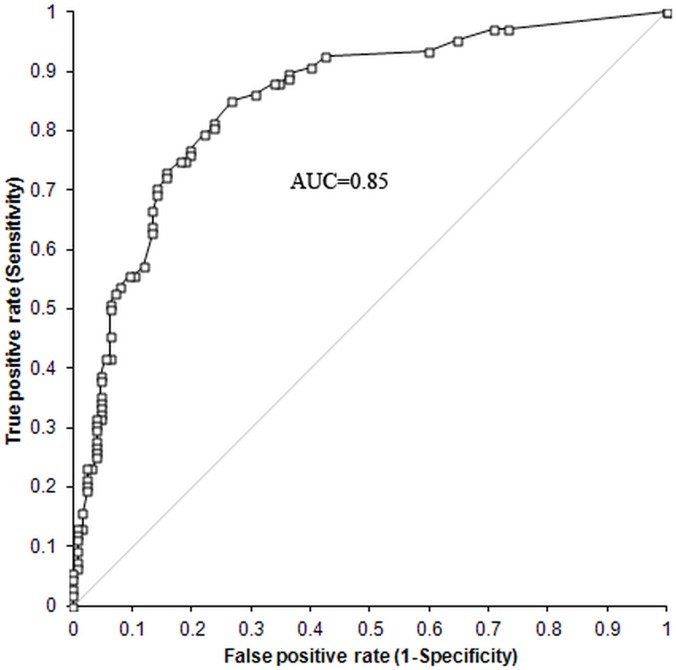
ROC curve of the predictive model for CHD. We developed a multivariate predictive model for CHD by means of forward logistic regression using as predictors the SNPs significant after controlling for FDR. We tested our model discrimination via the Hosmer_Lemeshow statistic and the receiver operating characteristic (ROC) curve with 95% CIs.

To search for a predictive model of CHD in the development population, the SNPs statistically significant after controlling for FDR both in the GWAS and in the candidate gene studies were entered together into multivariate forward logistic regression analysis. We obtained a predictive model with a good accuracy as indicated by a ROC AUC of 0.85 (95% CI 0.80–0.90) (Fig 3 and Table 2). The predictive model was developed in a population of 235 dogs, since 5 of the 240 individuals of the development cohort had missing genotypes. The logistic model combines 7 SNPs, namely BICF2P772455, BICF2G630227898, BICF2G630339806, BICF2G630558239, BICF2P548082, BICF2S230609 and BICF2S2452559 (Table 2). All the SNPs in the model have a β coefficient significantly different from zero, which indicates that all SNPs contribute to the predictive ability of the model (Table 2). The Se and Sp values of the model are shown in Table 3. As a general data, in the cut-off point showing the best equilibrium between Se and Sp, the model has a Se of 80% and a Sp of 78%.

**Table 2:** Predictive model for CHD.

**SNP**	**Chr: nt position**	**Notable nearby gene**	**nt change**	**Risk allele**	**Risk genotypes**	**β coefficient**	**OR (IC 95%)**	**p value**
BICF2P772455	chr4: 22691322	near to CHST3	A/G	G	GG	1.13	3.1 (1.3–7.5)	0.012
BICF2G630227898	chr20: 2704477	near to RAB7A	A/G	A	AA + AG	1.41	4.1 (1.8–9.2)	<0.001
BICF2G630339806	chr3: 40302288	near to CHSY1 and ADAMTS17	A/G	A	AA +AG	1.69	5.4 (1.9–15.0)	0.001
BICF2G630558239	chr7: 36171712	near to SMYD3	A/G	A	AA +AG	1.05	2.9 (1.4–5.8)	0.004
BICF2P548082	chr12: 16410934		A/G	G	GG + AG	0.81	2.2 (1.2–4.4)	0.017
BICF2S230609	chr18: 48695616	near to FGF4	A/G	A	AA	1.07	2.9 (1.5–5.7)	0.002
BICF2S2452559	chr10: 47923623	near to PKCE	A/G	G	GG +AG	0.79	2.2 (1.1–4.5)	0.033

The chromosomic position corresponds to CanFam 3.1.

**Table 3:** Predictive accuracy of the logistic model for Canine hip dysplasia.

**Predictive model for CHD**	
**Se (%)**	**Sp (%)**
93	58
80	78
70	86
42	95

Se: sensitivity; Sp: specificity

The dogs of the validation population were genotyped for the 7 SNPs included in the predictive model for CHD. A ROC curve was constructed with the predicted probability score for each subject of the validation cohort. A ROC curve with an AUC of 0.80 (95% CI 0.72–0.89) was found for the validation cohort. The predictive ability of the model was confirmed on a validation cohort. There were no statistical differences between the AUCs of the ROC curves of the two cohorts, 0.85 and 0.80, when compared using a two-sample Z test (Two-tailed p = 0.3161).

The relative contribution of each genetic variable to the predictive power of the model was calculated, BICF2G630227898 and BICF2G630339806 were the SNPs with a highest contribution, 21% and 20% respectively (S1 Fig).

One of the SNPs included in the model is BICF2P772455, which is located very close to the *CHST3* (carbohydrate chondroitin 6 sulfotransferase) gene, specifically, 14 bp upstream the initial ATG start codon. Among the 33 SNPs associated with CHD in the candidate gene analysis, BICF2P772455 was the SNP more strongly associated to the disease together a second SNP, BICF2P419109, located near the same gene, 1051 bp downstream *CHST3* (S2 Table). These two SNPs represented independent associations (LD r^2^<0.8). The *CHST3* gene encodes a sulfotransferase involved in chondroitin sulfate (CS) biosynthesis. CS is a structural component of the joint cartilage essential for its biomechanical properties, and so *CHST3* an interesting and promising candidate gene for CHD. Since in the public databases there are no SNPs described inside the *CHST3* canine gene, in order to know if there were other SNPs inside the *CHST3* gene more strongly associated to CHD, maybe a functional variant, we decided to sequence a chromosomic region of 5819 bp including the *CHST3* gene and its 5’ upstream and 3’ downstream flanking regions in 39 Labrador retrievers (20 unaffected and 19 affected individuals) (Labrador retriever *CHST3* Sequence NCBI accession number: JX402028.1). Apart from the 2 mentioned SNPs, we found other 29 SNPs located inside the *CHST3* gene (14 SNPs) and in its flanking regions (15 SNPs). However, none of the 29 SNPs was more strongly associated to CHD than BICF2P419109 and BICF2P772455 (data not shown).

## Discussion

CHD is an inherited disorder with a high prevalence in breeds of large size, such as Labrador retrievers, that ultimately leads to impaired mobility and function, and considerably reduces the quality of life of the dogs. Phenotype-based selective breeding of dogs has not been proven to be effective in significantly reducing the prevalence of CHD. The studies performed in the last recent years suggest that prediction of CHD is feasible using genetic markers. In this study we have developed a logistic regression model, based on genetic polymorphisms, with a good predictive accuracy for CHD susceptibility in Labrador retrievers, as indicated by a ROC-AUC of 85% [35]. The area under the ROC curve (AUC) is widely recognized as the measure of a diagnostic test's discriminatory power. The AUC can vary between 0.5 and 1. An area of 1 represents a perfect test while an area of 0.5 represents no discrimination. Predictive models with an AUC above 0.75 are considered as clinically useful and models with an AUC above 0.89 are considered as excellent. Logistic models with similar values of ROC-AUC as our model for CHD have been considered as highly predictive for some human diseases [23]. The predictive ability of the model was validated in an independent population of Labrador retrievers. This predictive model represents a significant advance in CHD early detection. It constitutes a useful tool for veterinarians to help them choose the most appropriate therapeutic approach once genetic predisposition is known. Therefore, a more individualized management of the disease might be achieved. It is also applicable in selection processes, since breeders could benefit from the information given by this predictive model as soon as a blood sample can be collected, and act accordingly.

The model for prediction of CHD includes 7 SNPs. The chromosomal region of 1 Mb surrounding the associated SNPs was analyzed using Ensembl database (www.ensembl.org) in search of potential candidate genes involved in CHD. Three of the seven SNPs lie within or near genes which codify proteins involved in extracellular matrix metabolism. The SNP BICF2P772455 is located in the 5’ UTR of the *CHST3* gene, just 14 bp upstream the initial ATG start codon. Thus, it could be part of a regulatory element affecting the transcription of the gene. The CHST3 enzyme is involved in chondroitin sulfate biosynthesis, specifically in the sulfation process. CS is an extracellular matrix component that plays an important role in cartilage function, providing this tissue with resistance and elasticity. Mutations in the *CHST3* gene have been previously found as associated with spondyloepiphyseal dysplasia in humans, which is a congenital skeletal development disorder characterized by joint dislocations, included hip dislocation [36,37]. A recent GWAS study in humans has reported that a SNP located in the *CHST11* gene, another gene of the same family as the *CHST3* gene, is associated with prevalence of hip OA [38]. Thus, we suggest that genes implicated in the CS synthesis could have a role in congenital canine and human disorders related to hip joint formation and that *CHST3* could be a potential credible gene implicated in CHD and secondary OA. The second SNP is BICF2G630339806 which is located near *CHSY1* (chondroitin sulfate synthase 1) and *ADAMTS17* (ADAM metallopeptidase with thrombospondin type 1 motif, 17) genes. As the *CHST3* gene, the CHSY1 enzyme also plays a critical role in the biosynthesis of CS. Studies in mice have suggested it to be an essential regulator of joint patterning [39], making a role for CHSY1 in CHD plausible. *ADAMTS17* is a member of the ADAMTS family of genes that encode secreted metalloproteases that modify extracellular structural proteins. Polymorphisms in ADAMTS genes have been associated with human diseases related with bone metabolism, including osteoporosis and OA [40,41], so ADAMTS17 could also be a potential gene implicated in CHD and secondary OA. The third SNP is BICF2S230609 located near the FGF4 (fibroblast growth factor 4) gene. Fibroblasts secrete the precursors of all the components of the extracellular matrix, maintaining the structural integrity of connective tissues. FGF family members possess broad mitogenic and cell survival activities and are implicated in embryonic development, morphogenesis and tissue repair, among other biological processes. Members of the FGF family have been proposed to control the limb bud outgrowth. Specifically, studies in mice suggested that FGF4 is implicated in embryonic distal limb morphogenesis, being responsible for the partial compensation of distal limb development in the absence of FGF8 [42]. Altogether, these results suggest that genes implicated in extracellular matrix synthesis/degradation could have a role in CHD. These results are in line with previous findings which indicate genes of extracellular matrix components as implicated in the pathogenesis of CHD [30], and point out the extracellular matrix metabolism as a key factor in CHD development.

Other two SNPs of the model lie very close to genes related to bone metabolism. The SNP BICF2G630227898 is located approximately 50 kb upstream the gene RAB7A which codifies for a small GTPase that is highly expressed and is predominantly localized at the ruffled border in bone-resorbing osteoclasts. During skeletal growth and remodeling the mineralized bone matrix is resorbed by osteoclasts, it has been described that downregulation of Rab7 impairs osteoclast polarization and bone resorption, underscoring the importance of Rab7 in osteoclast function and skeletal growth [43]. The gene PKCE, which codifies for the Protein Kinase C Epsilon, is situated approximately30 kb upstream the SNP BICF2S2452559. Protein Kinase Cs (PKCs) are a family of serine/threonine kinases involved in several cellular processes including cell proliferation, differentiation, apoptosis, and survival. Several studies have demonstrated that PKCs are involved in bone remodeling through regulation of osteoclast activity [44,45]. However, the exact role of individual PKC isoforms in the regulation of bone function is not fully clear. Our data agree with a recently published study performed with German Shepherd dogs [31], which has found SNPs associated to CHD located in close proximity to genes which are involved in bone formation and osteoclast activity, underlying the role of bone formation in CHD disease.

The gene SMYD3 (SET and MYND domain containing 3) is located about 0.5 Mb downstream of the SNP BICF2G630558239. SMYD3 is a histone methyltransferase that has been found to be implicated in muscle mass determination and skeletal muscle atrophy [46]. Thus, SMYD3 could be a potential candidate gene involved in the atrophy of hind leg muscles associated with CHD. Further research in this area is needed to confirm this hypothesis.

Finally, we have not found any potential candidate gene for CHD development located near the SNP BICF2P548082, this could be due to the fact that some of the genes near this SNP are still of unknown function or maybe because this SNP is in LD with a potential functional one in a distant genomic region.

Clearly, it is needed further research in this area to establish the link between the SNPs of the predictive model and the functional consequences for CHD and to clarify if the SNPs found are functional SNPs or SNPs in linkage disequilibrium with a nearby functional SNP.

In conclusion, we have developed a logistic model with a good accuracy for CHD prediction in Labrador retrievers based on the combination of 7 SNPs, several of them located near genes involved in extracellular matrix processes or bone metabolism. Our results in Labrador retrievers add evidence to the thought that genomics is the basis towards early detection of CHD. Whether our predictive model is valid or not for other dog breeds needs to be explored.

## Supporting Information

S1 Fig Relative contribution of each SNP to the predictive power of the model for CHD. (DOCX)

S1 Table SNPs significantly associated to CHD after correction for FDR in the GWAS analysis (p≤1.96x10-5). (XLSX)

S2 Table SNPs significantly associated to CHD after correction for FDR in the candidate gene study (p≤5x10-3). (XLSX)
