# Supplementary material for: A Genetic Predictive Model for Canine Hip Dysplasia: Integration of Genome Wide Association Study (GWAS) and Candidate Gene Approaches
Source: PLoS One. 2015 Apr 13;10(4):e0122558. doi: 10.1371/journal.pone.0122558 (PMC4395148; doi:10.1371/journal.pone.0122558)
Supplement: S1 Fig — (DOCX) [file pone.0122558.s001.docx]

**Figure S1.** Relative contribution of each SNP to the predictive power of the model for canine hip dysplasia.
